# Population difference gratings created on vibrational transitions by nonoverlapping subcycle THz pulses

**DOI:** 10.1038/s41598-021-81275-8

**Published:** 2021-01-21

**Authors:** Rostislav Arkhipov, Anton Pakhomov, Mikhail Arkhipov, Ihar Babushkin, Ayhan Demircan, Uwe Morgner, Nikolay Rosanov

**Affiliations:** 1 St. Petersburg State University, Saint Petersburg, Russian Federation; 2 ITMO University, Saint Petersburg, Russian Federation; 3 Ioffe Institute, Saint Petersburg, Russian Federation; 4 University of Hannover, Hannover, Germany; 5 Cluster of Excellence PhoenixD (Photonics, Optics, and Engineering—Innovation Across Disciplines), Hannover, Germany; 6 Max Born Institute, Berlin, Germany

**Keywords:** Physics, Optics and photonics, Optical physics, Terahertz optics

## Abstract

We study theoretically a possibility of creation and ultrafast control (erasing, spatial frequency multiplication) of population density gratings in a multi-level resonant medium having a resonance transition frequency in the THz range. These gratings are produced by subcycle THz pulses coherently interacting with a nonlinear medium, without any need for pulses to overlap, thereby utilizing an indirect pulse interaction via an induced coherent polarization grating. High values of dipole moments of the transitions in the THz range facilitate low field strength of the needed THz excitation. Our results clearly show this possibility in multi-level resonant media. Our theoretical approach is based on an approximate analytical solution of time-dependent Schrödinger equation (TDSE) using perturbation theory. Remarkably, as we show here, quasi-unipolar subcycle pulses allow more efficient excitation of higher quantum levels, leading to gratings with a stronger modulation depth. Numerical simulations, performed for THz resonances of the $H_20$ molecule using Bloch equations for density matrix elements, are in agreement with analytical results in the perturbative regime. In the strong-field non-perturbative regime, the spatial shape of the gratings becomes non-harmonic. A possibility of THz radiation control using such gratings is discussed. The predicted phenomena open novel avenues in THz spectroscopy of molecules with unipolar and quasi-unipolar THz light bursts and allow for better control of ultra-short THz pulses.

## Introduction

Generation of few-cycle THz pulses (0.1–10 THz) have attracted considerable interest over the past decades^1–4^ due to the growing number of applications in science and technology. For example, THz pulses are used for spectroscopy, since the THz range includes rotational and vibrational resonances of large molecules as well as transitions in various dielectrics and semiconductor structures^3–10^. They have a huge potential in other applications such as medicine, wireless communication systems, charge particles acceleration etc^3,4^. Nowadays, THz pulses up to subcycle duration became available^1–4^. Such durations can be much shorter than the relaxation times $T_1$ and $T_2$ of the resonant medium. That is, so called coherent interaction regime takes place, and effects of resonant light-matter interactions such as Rabi oscillations may arise^11^. If a resonant medium interacts coherently with a train of few-cycle pulses and the pulses do not overlap in the medium, population density gratings can be created in a two-level medium as it was shown in optical range using long nanosecond^12–14^, femtosecond and attosecond pulses^15–17^ as well as in THz range using multi-cycle THz^18^ pulses.

Such gratings are based on interaction between the pulses without an actual overlap, taking place indirectly, via oscillations of macroscopic polarization of the medium. The first pulse prepares the medium in a coherent superposition of the ground and excited states. This superposition exists within the time scale of $T_2$, until destroyed by decoherence. If a second pulse enters the medium within this time, it interacts with this induced atomic polarization, and in this way an inversion grating can be formed. This is in contrast to the traditional approach in which such gratings are generated as a result of interference of two or more long quasimonochromatic overlapping beams^19^.

The possibility of ultrafast creation and control of optical gratings by attosecond-long single-cycle and subcycle optical pulse trains was studied in^15–17^. However, generation of attosecond pulses with single-cycle and subcycle pulse duration requires a complicated setup. Besides, in the optical range, the field strengths needed to facilitate the effective interaction are extremely high ($~10^4-10^5$ ESU $\approx 10^6-10^7$ V/cm), which makes practical implementation even more complicated. In contrast, in the THz range, subcycle pulses can be generated much easier^1–4^.

Next, the pump power needed to realize coherent interactions in THz range is several orders of magnitude lower, provided that high THz vibrational transitions possess huge dipole moments $d_{12}$^20,21^. The latter allows using THz pulses experimentally available to date, and field strengths, significantly lower than in the optical range. It worth to note that practically available subcycle THz pulses often contain a burst of single polarity and a big tail of opposite polarity and small amplitude^1–4,22–38^. Action of such quasi-unipolar pulses is nearly the same as the true unipolar ones^38^.

Generation of unipolar and quasi-unipolar pulses and their applications in optics is a subject of active discussions^22–30^, see also recent review^31^ and references therein. These pulses, due to their unipolar character, effectively transfer their energy to charged particles, resulting in novel applications, including highly effective ionization and control of Rydberg atoms^32–34^, effective attosecond pulse generation^35^, effective excitation and ultra-fast control of electron wave packets dynamics^23,36–38^, charge acceleration^39^, holographic recording^40^ and others.

As we mentioned above, in previous works of the authors^15–17^ grating dynamics was studied in the optical range using few-cycle pulses in a two-level medium. However, due to broad spectral content of few-cycle pulses taking into account multilevel character of the system is of high importance. Some remarks on the possibility of grating formation in multi-level systems in the optical range were shortly reported in a prior work^16,41^. However, a detailed analysis of grating dynamics under such circumstances was up to now still missing. In this paper, we study a possibility of population density grating formation on vibrational THz resonance transitions in three- and multi-level media, assuming high values of transition dipole moments (tens to hundreds Debyes), created by subcycle THz pulses. We consider a possibility to use short quasi-unipolar subcycle pulses for grating formation, because they allow more effective excitation and control of quantum state population^23,36–38^. In previous studies, only bipolar few-cycle pulses were considered in this context^15,16^.

For the theoretical analysis of the multi-level system in question we use the standard perturbation approach for the time-dependent Schrödinger equation (TDSE) for “small” electric field amplitude. For numerical simulations, we use the Bloch equations for density matrix elements of a three-level medium. For analytical studies, we model the multi-level system as a quantum harmonic oscillator (HO) with equidistant levels assuming resonance frequency in THz range and high values of dipole moments of the resonant transitions. In spite of the fact, that our theoretical analysis is quite general, some particular examples will be mentioned.

To realize coherent light-matter interactions, the Rabi frequency $\Omega _R=d_{12}E_0/\hbar$ ($d_{12}$ is the transition dipole moment, $E_0$ is the electric field amplitude) should be larger than the inverse polarization relaxation time $T_2$, $\Omega _R > 1/T_2$ and the pulse duration $\tau _p <T_2$. First, most of molecules have vibration resonances in THz range^6–10^ and low energy states can be modelled, to some level of approximation, using quantum HO^42^. In our analysis below we consider a water molecule $H_2O$ having resonances in the interval 0.5–1 THz^43,44^. Other examples are Rydberg atoms with large quantum numbers $n>>1$—they also have large dipole moments^45^, can be arranged to possess long life times^46^ and resonance transitions in THz range^32–34,45^. Next, it is shown^47^ that the confinement potential of an electron in nanostructures and quantum dots (QD) for energy below the Fermi level is approximately parabolic, thus, the HO model can be used in this case. QD media can have extremely high values of transition dipole moments (tens-hundreds Debye) as well as $T_2$ at low temperatures as high as hundreds ns^48,49^; they also can be used for THz generation^50^. Furthermore, quantum cascade lasers can generate THz radiation^51^. Semiconductor nanostructures used in such devices have very high dipole moments (tens of Debye) and can operate as three-level systems^52,53^. In some media like crystals, containing impurities of rare-earth ions, the values of $T_2$ at low temperatures can approach extremely high values - from several seconds to several hours^54^.

There is another advantage of creating grating on vibrations transitions in THz range: In quantum HO in the first order perturbation approach, only the transition from the ground state to the 1st excited state is possible, in contrast to, for instance, 1/*r* potential. As we see here, this significantly reduces the probability of excitation of higher order levels, even if we go into nonperturbative regime with HO.

Furthermore, creation of the gratings on vibrational transitions in different systems opens novel opportunities in THz ultra-fast spectroscopy. For instance, diffraction of a weak probe pulse on such gratings can be used for spectroscopic measurements of $T_2$ – a task which is otherwise proved to be quite difficult experimentally^12–14^. As we show below, such gratings can be also used for control of THz radiation, for instance ultrafast pulse reshaping using fast mirrors.

This paper is organized as follows. First, in “Classical picture of grating formation in a system of classical HOs”, for a deeper understanding of the idea of grating formation, we consider aclassical picture of the gratings arising in a medium consisting of classical harmonic oscillators (HO). In “Multi-level vibration system: weak THz field strength” we consider a grating formation in a multi-level system using perturbation theory for TDSE, when the THz field strength is low. Here we use a $\delta$-pulse approximation, valid when the pulse duration is smaller than the inverse transition period of the medium. After this, pulses of finite duration are  considered. In “Numerical simulations” we perform numerical simulations for three-level HO using parameters for $H_2O$ molecule. A possibility of THz radiation control by the gratings is considered in the “Control of long THz pulses”. Finally, concluding remarks are drawn.

## Classical picture of grating formation in a system of classical HOs

**Figure 1:**
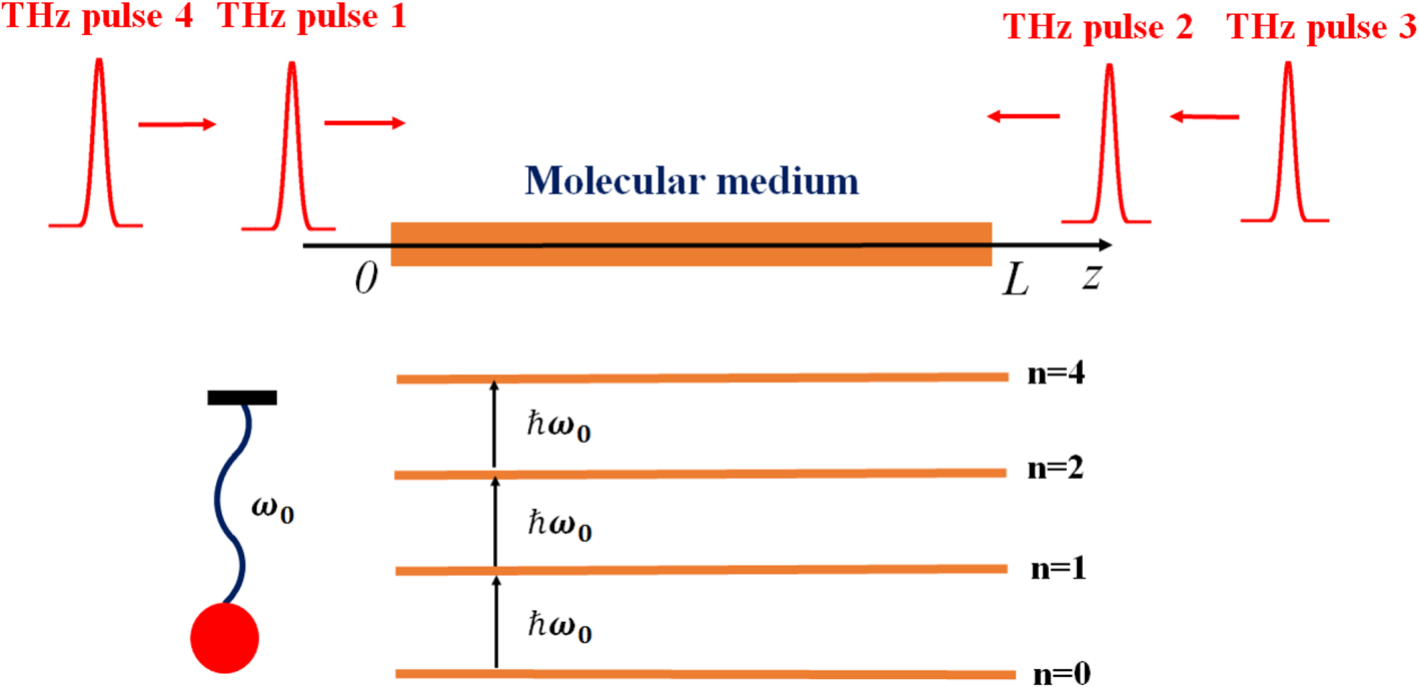
The simplest setup with four THz pulses counter-propagating in a medium to create a grating. Inset shows a 4-level HO molecular medium with the eigenfrequency $\omega _0$. This figure was created with Paint application for Windows and converted to eps using online convertor (https://image.online-convert.com/ru/convert-to-eps).

First, we consider the classical picture of a grating formation. Let a dielectric resonant medium consist of linear classical harmonic oscillators, non-interacting with each other and having eigen-frequency $\omega _0$, distributed along *z*-axis, see Fig. 1. The displacement of the oscillator at point *z* is governed by the following equation (neglecting damping of oscillations):

1 $$\begin{aligned} {\ddot{X}}(z,t) + \omega _0^2 X(z,t)=q/m E(z,t) \end{aligned}$$

Here *q* is the electron charge and *m* the oscillator mass. Let the medium be excited by a pair of extremely-short pulses with duration much smaller than the medium proper oscillations period $T_0=2\pi /\omega _0$. In this approximation, and for simplicity assuming the electric field in the form of two delta pulses $E(t)=S_0 \left[ \delta (t)+\delta (t-\Delta )\right]$, counter-propagating as shown in Fig. 1, with the delay $\Delta (z)$ depending on the oscillator position *z*. We assume low enough density of the oscillators on the string, i.e., changing of the pulse shape during the propagation is negligible. Under the action of such kick-like excitation, the medium starts to harmonically oscillate at the eigen-frequency $\omega _0$. It can be easily shown that the response of the medium can be represented as^55^

2 $$\begin{aligned} X(z,t)&= {} X_0 \sin \omega _0t, t<\Delta , \nonumber \\&= {} X_0 \sin \omega _0t+ X_0 \sin (\omega _0(t-\Delta )) =X_0\cos \left( \omega _0\Delta /2\right) \sin \left( \omega _0t+\omega _0\Delta /2\right) , t>\Delta . \end{aligned}$$

From the last expression it is seen that after arrival of the second pulse, the amplitude of the displacement periodically depends on the pulse-to-pulse delay $~\cos \left( \omega _0\Delta /2\right)$. This can be interpreted as periodic grating formation in the system of classical oscillators. In the next section, we consider the corresponding quantum picture.

## Multi-level vibration system: weak THz field strength

### Theoretical approach

In the previous studies we predicted population density gratings formation in a two-level medium^15–17^. However, subcycle pulses have broadband spectra and two-level approximation can be inapplicable. In this section we consider a multi-level vibration system, namely, a quantum harmonic oscillator (HO). The HO model is commonly used as a simplest model for theoretical description of molecular vibrations^42^.

Interaction of a subcycle pulse with a quantum system with arbitrary configuration of states is governed by the TDSE describing the evolution of the wave function $\psi$^42^:

3 $$\begin{aligned} i\hbar \frac{\partial \psi }{\partial t} = \Big [ {{\hat{H}}}_0 + V(t) \Big ] \psi . \end{aligned}$$

${{\hat{H}}}_0$ is the intrinsic Hamiltonian of the unperturbed system, and $V(t)=-qxE(t)$ is the interaction potential with the excitating pulse in the dipole approximation. The wave functions of the eigenstates are the eigenfunctions of the intrinsic Hamiltonian ${{\hat{H}}}_0$ and given by^42^:

4 $$\begin{aligned} \psi _n(x) = \Big (\frac{m\omega _0}{\pi \hbar } \Big )^{1/4} \frac{1}{\sqrt{2^n n!}} e^{-\frac{m \omega _0 x^2}{2 \hbar }} H_n\Big ( x \sqrt{\frac{m \omega _0}{\hbar }}\Big ), \end{aligned}$$

where $H_n\Big (x\sqrt{\frac{m \omega _0}{\hbar }}\Big )$ - Hermite polynomials of order *n*. The general solution of the TDSE (3) can be written as a superposition of eigenstates $\psi _n$ with the amplitudes $a_n$:

5 $$\begin{aligned} \psi (x,t)=\sum _n a_n(t) \psi _n(x). \end{aligned}$$

Let us assume that the system was initially in the ground state with $n=0$ and the pulse amplitude is small. The amplitudes of the eigenstates can be easily calculated in the 1st order of the perturbation theory^42^:

6 $$\begin{aligned} a^{I}_{n}=-\frac{i}{\hbar }\int V_{0n}e^{i\omega _{0}t}dt. \end{aligned}$$

The transition probability of a HO from the ground state of the discrete spectrum to the *n*-th state $w_{0n}$ is given by $|a^{I}_{n}|^2$^42^:

7 $$\begin{aligned} w_{0n}=\frac{1}{\hbar ^2} \Big | \int V_{0n}e^{i\omega _{0}t}dt \Big |^2. \end{aligned}$$

$V_{0n}$ is the matrix element of the perturbation operator, which is expressed through the matrix element of the dipole moment $d_{n,n+1}$ as:

$$\begin{aligned} V_{n,n+1} = - d_{n,n+1} E(t). \end{aligned}$$

For HO, the matrix $d_{n,n+1}$ is given by^42^:

8 $$\begin{aligned} d_{n,n+1}= q \sqrt{\frac{\hbar n(n+1)}{2m\omega _0}}. \end{aligned}$$

Other matrix elements are zero, because in HO only transitions between neighbouring states are possible. That is, in the 1st order perturbation approach, only transition from the ground state to the 1st excited state has nonzero probability, i.e.,

9 $$\begin{aligned} w_{01}=\frac{q^2}{2m\omega _0\hbar } \Big | \int E(t)e^{i\omega _{0}t}dt \Big |^2. \end{aligned}$$

This allows us to consider HO as an effective two-level medium, as least in perturbative approximation. This gives rise to high-contrast grating formation only on the first transition $0-1$ of the vibrational spectrum.

### Population density grating formation and their control using $\delta$-pulses

Before providing detailed analysis of the grating dynamics let us consider for simplicity interaction of a HO with a $\delta$-pulse. A validity of this approximation follows from the fact that experimentally obtained subcycle pulse shape contains a short burst of electric field (half-wave) of one polarity and a long damped tail of small amplitude of the opposite polarity^1–4,22–30^. In view of the small amplitude of the tail, the main contribution is given by the strong half-wave part^38^. Assuming, that the pulse duration is smaller than all resonant transition periods in the atom/molecule, we can describe our driving field as a set of delta-function-like pulses:

10 $$\begin{aligned} E(t)=\sum _n S_{E,n}\delta (t-\Delta _{n}), \end{aligned}$$

having the time delays $\Delta _n$ and electric pulse area $S_{E,n}=\int E_n(t)dt$^56^, $E_n$ is the electric field of the *n*-th pulse.

In the case of a spatially extended medium, one can consider a string of atoms distributed along the *z* axis. If the concentration of atoms in such a string is relatively small, one may neglect nonlinear pulse shape modification due to propagation effects. A setup for a grating formation in the case with four pulses is shown in Fig. 1 (similar scheme was used earlier for a two-level medium^15,16^). The first pulse propagates in the medium from left to right, whereas the second one travels from right to left, after the 1st one already left the medium, so that the pulses do not overlap in the medium. Under these conditions, the delay between two pulses is constantly changing with time. The 3rd pulse propagates after the 2nd one in the same direction. The 4th pulse travels in the same direction as the 1st one after 3rd pulse left the medium. To track this situation without directly solving the wave equation, we may consider every point separately. In such consideration, the difference from one point to another in *z*-direction is mapped to the delay between two pulses $\Delta \sim z/c$. Thus, we can consider a single-atom response and calculate the populations of the levels in dependence on the delay between two ultra-short pulses in order to show the existence of a grating as well as to estimate its properties.

First, consider the action of two pulses propagating in the opposite directions as shown in Fig. 1:

11 $$\begin{aligned} E(t)=S_0 \left[ \delta (t) + \delta (t-\Delta )\right] , \end{aligned}$$

where $\Delta$ is the delay between two pulses. Using Eq. (7) and performing integration it is easy to obtain an expression for the transition probability $w_{01}$ (the only nonzero one, according to Eq. (8)):

12 $$\begin{aligned} w_{01}=2\frac{d^2_{01}S^2_{0}}{\hbar ^2} \left( 1+\cos \omega _{01} \Delta \right) . \end{aligned}$$

From Eq. (12), the periodic dependence of the transition probability on the delay between the pulses $\Delta$ is seen. Since in the case of an extended medium, the delay $\Delta \sim z/c$ determines the time of arrival of the second pulse to a point of the medium with coordinate *z*, Eq. (12) shows a possibility of a periodic grating formation in the medium. Equation (12) is valid for any multi-level quantum system, when the concentration of particles is small and the change in the pulse shape during the propagation process can be neglected. However, formula Eq. (12) was obtained in the perturbation theory in the weak-field approximation. The case of an arbitrary strong driving field will be considered below.

We now show a possibility of erasing and spatial frequency multiplication of the gratings. Suppose that the system interacts with three pulses, see Fig. 1:

13 $$\begin{aligned} E(t)=S_0 \left[ \delta (t)+\delta (t-\Delta )+ \delta (t-\Delta -\Delta _{23})\right] , \end{aligned}$$

where $\Delta _{23}$ is the delay between the 2nd and the 3rd pulse. Using Eq. (7) for the transition probability it is easy to obtain

14 $$\begin{aligned} w_{01}=\frac{d^2_{01}S^2_{0}}{\hbar ^2} |1+e^{i\omega _{01}\Delta } + e^{i\omega _{01}\Delta }e^{i\omega _{01}\Delta _{23}}|^2. \end{aligned}$$

From Eq. (14) it is seen that when $e^{i\omega _{01}\Delta _{23}}=-1$, the 3rd pulse erases the grating since the probability of transition does not depend on the delay and thus on the spatial position.

Next, let the system interact with the 4th pulse (see Fig. 1):

15 $$\begin{aligned} E(t)=S_0 \left[ \delta (t)+\delta (t-\Delta )+ \delta (t-\delta -\Delta _{23}) + \delta (t-\Delta _{23}-\Delta _{34})\right] , \end{aligned}$$

where $\Delta _{34}$ - delay between the 3rd and the 4th pulse. For the transition probability we obtain:

16 $$\begin{aligned} w_{01}=\frac{d^2_{01}S^2_{0}}{\hbar ^2} |1+e^{i\omega _{01}\Delta } + e^{i\omega _{01}\Delta }e^{i\omega _{01}\Delta _{23}} + e^{i\omega _{01}\Delta }e^{i\omega _{01}\Delta _{23}}e^{i\omega _{01}\Delta _{34}}|^2. \end{aligned}$$

We now show the possibility of the multiplication of the spatial frequency of the gratings analogously as it is done in a two-level system^15^. We choose the following values of the delays between pulses: $\Delta _{23}=\pi /\omega _{01} +2\pi k /\omega _{01}$, that is $e^{i\omega _{01}\Delta _{23}}=-1)$ and $\Delta _{34}=\Delta +2\pi k / \omega _{01}$, where $k=0,2,4,6$ Hence, from Eq. (16) it is easy to obtain:

17 $$\begin{aligned} w_{01}=\frac{d^2_{01}S^2_{0}}{\hbar ^2} |1-e^{2i\omega _{01}\Delta }|^2 = 2\frac{d^2_{01}S^2_{0}}{\hbar ^2} \left( 1 - \cos 2\omega _{01}\Delta \right) . \end{aligned}$$

From Eq. (17) it is seen that the spatial frequency of the gratings is multiplied by a factor of two. Thus, we showed that by selecting the delay between the pulses it is possible to control period of the multi-level-based grating.

### Population density grating formation using subcycle THz pulses of finite duration

**Figure 2:**
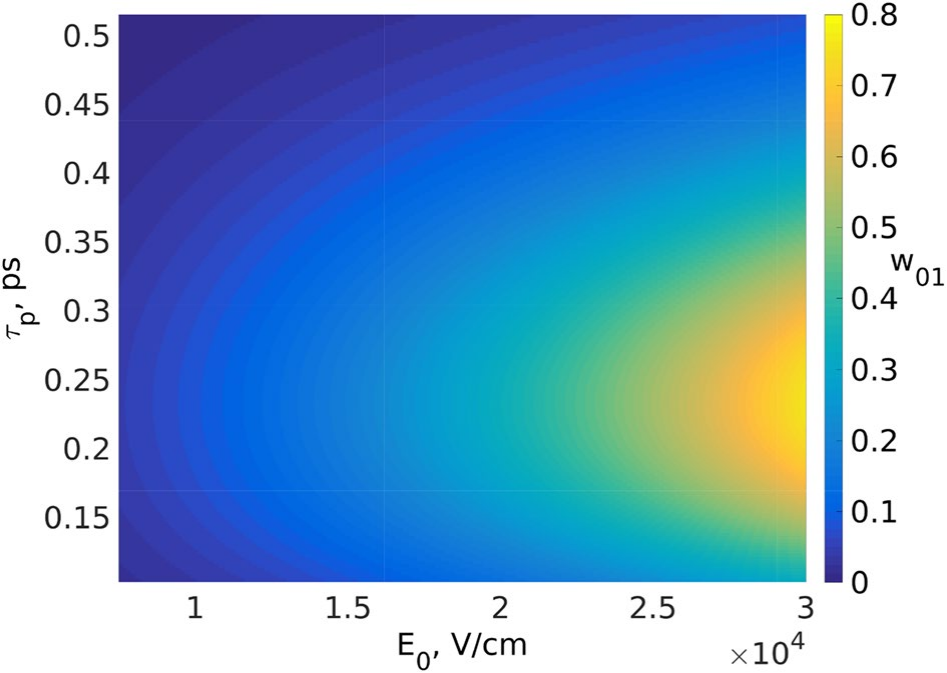
Dependence of the population of the 1st excited state $w_{01}$ on the pulse duration $\tau _p$ and the amplitude $E_0$ calculated for $H_2O$ molecule. Other parameters: $\Delta =3$ ps, $\omega _0/2\pi =0.97$ THz, $d_{01}=19.2$ Debye. This figure was created with Matlab R2016b (www.mathworks.com).

Using Eq. (7), we show a possibility of existence of population density gratings in HO-type systems for the pulses of finite duration. As we mentioned above, experimentally obtained half-cycle THz pulses contain a strong half-wave of high amplitude and a long weak tail of the opposite polarity^1–4^. It was shown in^38^, that the impact of this tail can be neglected, provided that its amplitude is small, and duration is larger than the width of the main half-wave. Hence, we first neglect for simplicity this tail and consider the impact on the system of two Gaussian pulses given by:

18 $$\begin{aligned} E(t)=E_0\exp \left( -{t}^2/\tau _p^2\right) + E_0\exp \left( -(t-\Delta )^2/\tau _p^2\right) , \end{aligned}$$

where $\Delta$ is the time delay between two pulses. In the subsequent analysis we include the tail into consideration. Substituting Eq. (18) into Eq. (7) and taking account that $\int e^{ibx-ax^2}dx=\sqrt{\frac{\pi }{a}}e^{\frac{-b^2}{4a}}$, we obtain

19 $$\begin{aligned} w_{01}=\frac{2 \pi q^2 E_0^2\tau _p^2}{m\hbar \omega _0}\exp \frac{-\omega _0^2\tau _p^2}{2}\left( 1+\cos \omega _0\Delta \right) , \end{aligned}$$

where we have explicitly used Eq. (8) for $d_{01}$ as well as denoted the transition frequency $0 \rightarrow 1$ as $\omega _0 = \omega _{01}$. Expression Eq. (19) is proportional to the square of the field strength $E_0$ and at the first glance it seems that probability $w_{01}$ can be very high at large field amplitudes. However, Eq. (19) was obtained using perturbation approach and is valid when the electric field amplitude is small, i.e. $q^2E_0^2\pi \tau _p^2/m \ll \hbar \omega _0$. From Eq. (19) one can also see that the periodic dependence of the transition probability (population of the first excited state) on the delay between pulses $\Delta$ is similar to the case of $\delta$-function-like pulses considered earlier. The modulation depth of the grating is proportional to the square of the electric field amplitude of the pulse. Formula Eq. (19) can be interpreted as a harmonic inversion grating created by a pair of counter-propagating subcycle pulses in a spatially-extended medium. Thus, in the case of the pulses with finite duration the existence of the inversion gratings is also possible.

We apply the theory developed above to a $H_2O$ molecule. It has isolated resonances in the THz range, in particular, around 0.97-0.99 THz according to experimental results of^43^. Below we take $\omega _0/2\pi \approx 0.97$ THz . Using the value for the “characteristic radius” of water $R=4$ Angstrom^44^, we estimate transition dipole moment as $d_{01}=qR=19.2$ Debye (*q* is the electron charge). The two-dimensional diagram in Fig. 2 illustrates the dependence of the 1st excited state population $w_{01}$ Eq. (19) for water molecules vs the pulse duration $\tau _p$ and the pulse amplitude $E_0$ for $\Delta =3$ fs. The maximal probability value $w_{01}$ at the fixed delay $\Delta$ is determined by the term $\tau _p^2\exp \frac{-\omega _0^2\tau _p^2}{2}$. For small pulse durations (shorter than the transition period, $\omega _0\tau _p \ll 1$) this term increases with increase of $\tau _p$, reaching its maximum value at certain point. With the particular parameters mentioned above, $w_{01}$ reaches its maximum at the pulse duration $\tau _p$ of around 200 fs. When the pulse duration becomes larger than the transition period, $\omega _0\tau _p \gg 1$, the population $w_{01}$ tends to zero. It means, that an unipolar pulse with a duration smaller than the inverse transition frequency ($\omega _0\tau _p \ll 1$) acts more effectively than a long unipolar one with a larger duration and the same amplitude. This statement is valid in general case of quasi unipolar ones, see below.

It can be also seen, that the modulation depth of the gratings significantly depends on the duration of the incident pulses and their amplitudes. It is also seen from Fig. 2 that for creation of gratings, electric fields of small amplitude of $\sim$ kV/cm are sufficient, what can be easily reached in practice^1–4^.

Since the HO model is used to describe molecular vibrations (more precisely, low excited vibration states), and the oscillation frequencies of the molecules could lie in the THz region, the analytical result Eq. (19) clearly shows the possibility of a grating creating in molecular systems using a pair of THz subcycle pulses with experimentally available strengths, several orders of magnitude lower than in the optical range.

Now, we proceed further to take into account the tails of the Gaussian pulses. We assume, instead of Eq. (18), the following more general expression for the exciting field:

20 $$\begin{aligned} E(t)=E_0\exp \left( -{t}^2/\tau _p^2\right) \cos (\Omega t + \varphi _1) + E_0\exp \left( -(t-\Delta )^2/\tau _p^2\right) \cos (\Omega (t-\Delta ) + \varphi _2), \end{aligned}$$

with the carrier-wave frequency $\Omega$ and the carrier-envelope phase (CEP) $\varphi _i$. For clarity, we assume below CEP for all pulses in the sequence to be fixed $\varphi _i = \varphi = \text {const}$, i.e., that the pulses are coming from a CEP-stabilized laser pulse source. The electric pulse area for such pulses is given as:

21 $$\begin{aligned} S_E = \sqrt{\pi } E_0 \tau _p \exp \left( - \Omega ^2 \tau _p^2 / 4\right) \cos \varphi , \end{aligned}$$

so that these pulses can be safely treated as unipolar ones if $\Omega \tau _p \sim 1$ or even $\Omega \tau _p \ll 1$ and $\varphi \ne \pm \pi /2$.

Substituting now Eq. (20) into Eq. (7) and performing integration we find:

22 $$\begin{aligned} w_{01}=\frac{\pi q^2 E_0^2\tau _p^2}{m\hbar \omega _0} \exp \frac{- (\omega _0^2 + \Omega ^2) \tau _p^2}{2} \Big ( \cosh \left( \omega _0 \Omega \tau _p^2\right) + \cos 2 \varphi \Big ) \left( 1+\cos \omega _0\Delta \right) . \end{aligned}$$

**Figure 3:**
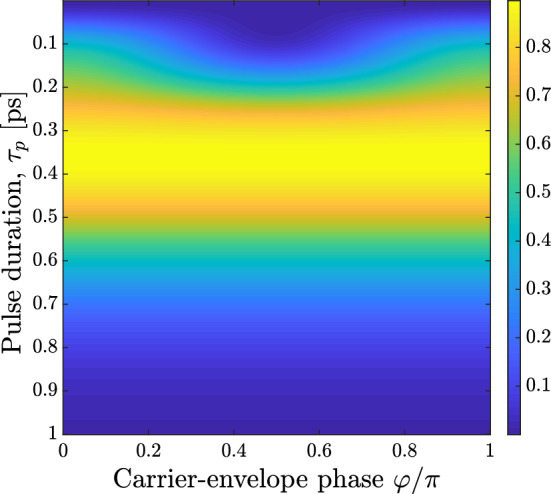
Dependence of the parameter $\kappa$ from Eq. (23) on the carrier-envelope phase (CEP) $\varphi$ and the pulse duration $\tau _p$; other parameters: $\omega _0/2\pi = 0.97$ THz, $\Omega = 2 \cdot 10^{12}$ $\hbox {s}^{-1}$. This figure was created with Matlab R2018a (www.mathworks.com).

Equation (22) again yields a periodic dependence of the transition probability $w_{01}$ (population of the first excited state) on the delay $\Delta$ similarly to Eq. (19) above. Moreover, Eq. (19) contains some interesting peculiarities. When CEP $\varphi = \pm \pi /2$, i.e. the exciting pulses are strictly bipolar according to Eq. (21) and $S_E = 0$, the population gratings still arise, even though they have smaller modulation depth. The dependence of the modulation amplitude on the duration and CEP of the pump pulses is determined from Eq. (22) by the following factor:

23 $$\begin{aligned} \kappa = \omega _0^2 \tau _p^2 \exp \frac{- (\omega _0^2 + \Omega ^2) \tau _p^2}{2} \Big ( \cosh \left( \omega _0 \Omega \tau _p^2\right) + \cos 2 \varphi \Big ), \end{aligned}$$

where we have introduce the factor $\omega _0^2$ to make the parameter $\kappa$ dimensionless. Figure 3 illustrates the dependence of this parameter on CEP $\varphi$ and the pulse duration $\tau _p$. One can see that $\kappa$ achieves its maximal value at $\tau _p \approx 0.4$ ps and $\cos \varphi = 1$. Also, it is worth noting that in the limit $\Omega \rightarrow 0$ Eq. (22) coincides with Eq. (12).

It is also seen from Fig. 3 that the grating modulation depth is larger for subcycle pulses ($\Omega \tau _p \ll 1$) than for multi-cycle ones, and it is smaller for bipolar pulses (when $\varphi =\pi /2$ and the pulse area $S_E=0$). This fact is in agreement with our previous results, which showed that quasi-unipolar subcycle pulses allow more efficient nonresonant excitation of quantum systems with respect to single-cycle and multi-cycle ones^37,38^.

Let us check now the results of the previous section for the sequence of three and four pump pulses. For three pulses we have the exciting electric field in the form:

$$\begin{aligned} E(t)&= {} E_0\exp \left( -{t}^2/\tau _p^2\right) \cos (\Omega t + \varphi ) + E_0\exp \left( -(t-\Delta )^2/\tau _p^2\right) \cos (\Omega (t-\Delta ) + \varphi )\\& \quad +E_0\exp \left( -(t - \Delta - \Delta _{23})^2/\tau _p^2\right) \cos (\Omega (t-\Delta - \Delta _{23}) + \varphi ), \end{aligned}$$

and Eq. (7) gives:

24 $$\begin{aligned} w_{01}&= {} \frac{\pi q^2 E_0^2\tau _p^2}{2 m\hbar \omega _0} \exp \frac{- (\omega _0^2 + \Omega ^2) \tau _p^2}{2} \Big ( \cosh \left( \omega _0 \Omega \tau _p^2\right) + \cos 2 \varphi \Big ) \nonumber \\\times & {} \Big ( 3 + 2 \cos \omega _0\Delta + 2 \cos \omega _0 (\Delta + \Delta _{23}) + 2 \cos \omega _0\Delta _{23} \Big ). \end{aligned}$$

If we choose $\Delta _{23}=\pi /\omega _{0} +2\pi n \omega _{0}$, Eq. (24) reduces to:

$$\begin{aligned} w_{01}=\frac{\pi q^2 E_0^2\tau _p^2}{2 m\hbar \omega _0} \exp \frac{- (\omega _0^2 + \Omega ^2) \tau _p^2}{2} \Big ( \cosh \left( \omega _0 \Omega \tau _p^2\right) + \cos 2 \varphi \Big ), \end{aligned}$$

i.e., the third pulse erases the population grating and we get a constant value of $w_{01}$ along the medium. Finally, when we add the fourth pulse:

$$\begin{aligned} E(t)&= {} E_0\exp \left( -{t}^2/\tau _p^2\right) \cos (\Omega t + \varphi ) + E_0\exp \left( -(t-\Delta )^2/\tau _p^2\right) \cos (\Omega (t-\Delta ) + \varphi )\\& \quad + E_0\exp \left( -(t - \Delta - \Delta _{23})^2/\tau _p^2\right) \cos (\Omega (t-\Delta - \Delta _{23}) + \varphi ) \\& \quad + E_0\exp \left( -(t - \Delta - \Delta _{23} - \Delta _{34} )^2/\tau _p^2\right) \cos (\Omega (t-\Delta - \Delta _{23} - \Delta _{34}) + \varphi ), \end{aligned}$$

and we obtain with Eq. (7):

25 $$\begin{aligned} w_{01}= & {} \frac{\pi q^2 E_0^2\tau _p^2}{2 m\hbar \omega _0} \exp \frac{- (\omega _0^2 + \Omega ^2) \tau _p^2}{2} \Big ( \cosh \left( \omega _0 \Omega \tau _p^2\right) + \cos 2 \varphi \Big ) \Big (4 + 2 \cos \omega _0\Delta + 2 \cos \omega _0 (\Delta + \Delta _{23})\nonumber \\&+ 2 \cos \omega _0\Delta _{23} + 2 \cos \omega _0 (\Delta + \Delta _{23} + \Delta _{34}) + 2 \cos \omega _0 (\Delta _{23} + \Delta _{34}) + 2 \cos \omega _0\Delta _{34} \Big ). \end{aligned}$$

As we choose the delays between pulses: $\Delta _{23}=\pi /\omega _{0} +2 \pi n/ \omega _{0}$ and $\Delta _{34} = \Delta + 2\pi n/ \omega _{0}$ with integer *n*, we get the following population grating:

26 $$\begin{aligned} w_{01}=\frac{\pi q^2 E_0^2\tau _p^2}{m\hbar \omega _0} \exp \frac{- (\omega _0^2 + \Omega ^2) \tau _p^2}{2} \Big ( \cosh \left( \omega _0 \Omega \tau _p^2\right) + \cos 2 \varphi \Big ) \left( 1-\cos 2 \omega _0\Delta \right) , \end{aligned}$$

i.e., the spatial frequency of the grating is multiplied by factor of 2.

## Numerical simulations

To check our analytical theory and observe, what happens in the nonperturbative regime, we solve numerically a system of Bloch equations for density-matrix element, which models interaction of a three-level medium with THz pulses:

27 $$\begin{aligned} \frac{\partial }{\partial t} \rho _{21}= & {} -\rho _{21}/T_{21}-i \omega _{21} \rho _{21} - i \frac{ d_{21} E}{\hbar } ( \rho _{22} - \rho _{11} ) - i \frac{ d_{13} E}{\hbar } \rho _{32} + i \frac{ d_{23} E}{\hbar } \rho _{31}, \end{aligned}$$

28 $$\begin{aligned} \frac{\partial }{\partial t} \rho _{32}= & {} -\rho _{23}/T_{32} -i \omega _{32} \rho _{32} - i \frac{ d_{32} E}{\hbar } ( \rho _{33} - \rho _{22} ) - i \frac{ d_{21} E}{\hbar } \rho _{31} + i \frac{ d_{13} E}{\hbar } \rho _{21}, \end{aligned}$$

29 $$\begin{aligned} \frac{\partial }{\partial t} \rho _{31}&= {} -\rho _{31}/T_{31} -i \omega _{31} \rho _{31} - i \frac{ d_{31} E}{\hbar } ( \rho _{33} - \rho _{11} ) - i \frac{ d_{21} E}{\hbar } \rho _{32} + i \frac{ d_{23} E}{\hbar } \rho _{21}, \end{aligned}$$

30 $$\begin{aligned} \frac{\partial }{\partial t} \rho _{11}&= \rho _{22}/T_{22}+\rho _{33}/T_{33}+{} i \frac{ d_{21} E}{\hbar } ( \rho _{21} - \rho _{21}^* ) - i \frac{ d_{13} E}{\hbar } (\rho _{13} - \rho _{13}^*), \end{aligned}$$

31 $$\begin{aligned} \frac{\partial }{\partial t} \rho _{22}&= {} -\rho _{22}/T_{22} -i \frac{ d_{21} E}{\hbar } ( \rho _{21} - \rho _{21}^* ) - i \frac{ d_{23} E}{\hbar } (\rho _{23} - \rho _{23}^*), \end{aligned}$$

32 $$\begin{aligned} \frac{\partial }{\partial t} \rho _{33}&= {} -\rho _{33}/T_{33}+ i \frac{ d_{13} E}{\hbar } ( \rho _{13} - \rho _{13}^* ) + i \frac{ d_{23} E}{\hbar } (\rho _{23} - \rho _{23}^*). \end{aligned}$$

Equations  (27)–(29) describe the evolution of off-diagonal elements of the density matrix $\rho _{21}, \rho _{32}, \rho _{31}$, which are associated with the polarization of the medium. Equations  (30)–(32) describe the evolution of diagonal elements of the density matrix $\rho _{11}$, $\rho _{22}$, $\rho _{33}$, which have the meaning of populations of the first, second and third levels, correspondingly. Parameters $d_{21}, d_{23}, d_{13}$ are the dipole moments of the corresponding transitions, $\omega _{21}$, $\omega _{32}$, $\omega _{31}$ are the transition frequencies. In our case, we consider a three-level HO medium, so $\omega _{21}=\omega _{32}=\omega _0$ and $\omega _{31}$=$2\omega _0$, $d_{13}=0$. Equations  (27)–(29) also contain relaxation times of the non-diagonal elements of the density matrix $T_{21}$, $T_{32}$, $T_{31}$, and population lifetimes of the 2nd and 3rd levels respectively $T_{22}$ and $T_{33}$. We performed series of numerical simulations for both low and high THz field amplitudes.

### Low-power THz fields

Equation (19) was obtained using perturbation approach valid for low field strength. In the 1st order perturbation approach, only probability transition $w_{01}$ is non-vanishing. This is because transition dipole moments in HO $d_{0,n}=0$ for *n* larger than 1. Numerical simulations (see below) showed that populations of higher levels are 10-100 times lower than the populations of the 2nd level $w_{01}$. This fact enables the possibility of creating the high contrast grating on the main transition $0 \rightarrow 1$ neglecting other transitions using weak THz field values.

**Figure 4:**
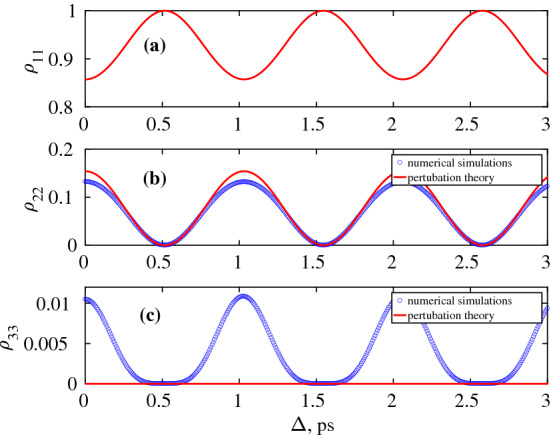
Dependence of the populations $\rho _{11}$ (**a**), $\rho _{22}$ (**b**), and $\rho _{33}$ (**c**) on the delay $\Delta$ between two Gaussian THz pulses for the $H_2O$ molecule. Blue circles show the result of the numerical simulations, red solid line show the analytical results obtained from Eq. (19). Parameters: $E_0=15 000$ V/cm, $\tau _p=150$ fs, $d_{12}=19.2$ Debye, $\omega _0/2\pi =0.97$ THz, all the relaxation times $T_{ik}=\infty$. This figure was created with Matlab R2016b (www.mathworks.com).

To confirm this statement, we performed numerical simulations using system of density matrix equations  (27)–(32) with a pair of Gaussian pulses  (18). Figure 4 shows the behaviour of the populations $\rho _{11}$ (a), $\rho _{22}$ (b), and $\rho _{33}$ (c) versus the delay $\Delta$ for the water molecule and $E_0=15$ kV/cm assuming infinite relaxation times. The influence of finite relaxation times will be studied in the next section. It can be seen from Fig. 4b that the grating obtained numerically has the same shape and roughly the same amplitude as predicted by Eq. (19). Besides, the population of the 3rd level $\rho _{33}$ is 10 times smaller than $\rho _{32}$.

### High-power THz fields

**Figure 5:**
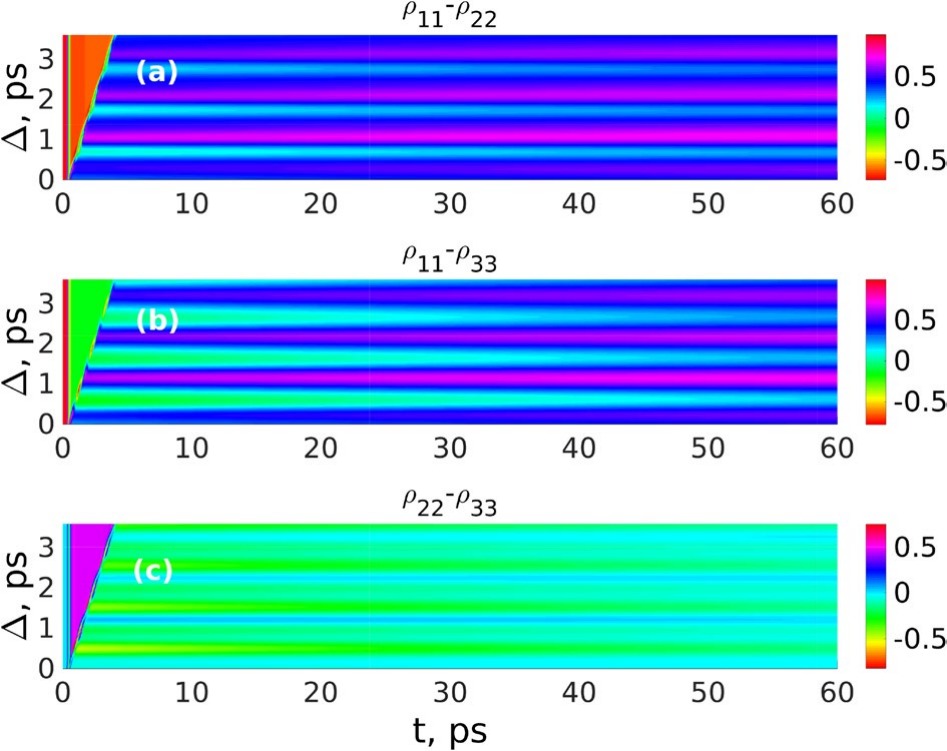
Dependence of the populations difference $\rho _{11}-\rho _{22}$ (**a**), $\rho _{11}-\rho _{33}$ (**b**), $\rho _{22}-\rho _{33}$ (**c**) on the delay $\Delta$ between two Gaussian THz pulses and time *t* for parameters of the $H_20$ molecule ($\omega _0/2\pi =0.97$ THz, $d_{12}=19.2$ Debye). Other parameters: $E_0= 150$ kV/cm, $\tau _p=150$ fs, $T_{22}=T_{33}=250$ ps, $T_{21}=T_{32}=T_{31}=5$ ps. This figure was created with Matlab R2016b (www.mathworks.com).

To study the grating dynamics in the high-power field we performed a series of the numerical simulations of the Bloch equations described above at different pump field strengths and durations. Figure 5 shows the behaviour of the populations difference $\rho _{11}-\rho _{22}$ (a), $\rho _{11}-\rho _{33}$ (b), $\rho _{22}-\rho _{33}$ (c) versus the delay $\Delta$ between two Gaussian THz pulses and time *t* for the $H_20$ molecule ($\omega _0/2\pi =0.97$ THz, $d_{12}=19.2$ Debye) for the following parameters: $E_0=150$ kV/cm, $\tau _p=150$ fs. Typical lifetimes of the vibrational levels $T_1$ and coherence times $T_2$ of vibration states are in the ps range^57,58^. We take relaxation population lifetimes $T_{22}=T_{33}=250$ ps and coherence lifetimes $T_{21}=T_{32}=T_{31}=5$ ps^57,58^.

One can see noticeable differences to the case of low field strength described above. The grating shape is not harmonic anymore, and has complex peak structure in contrast to the weak-field case. Also in contrast to the case of the two-level system, where both analytical and numerical solutions predict harmonic shape only^15,16^. Next, due to finite values of the relaxation times, the grating modulation depth decreases dramatically with time.

To summarize the results of these sections, we see that using THz pulses and vibrational transitions introduces many differences and advantages with respect to the previously studied case of optical field excitation. It allows reducing field strength using experimentally available subcycle THz fields. One can use only the  transition $0 \rightarrow 1$ for grating formation in the weak-field case. The shape of the grating can be controlled by increasing the field strength, in contrast to the case of a two-level medium, where grating shape was always harmonic.

## Control of long THz pulses

In this section, we describe an application of the gratings described above, to control propagation of THz pulses. As it follows from the Bloch equations, the modification of polarization in the linear regime corresponding to a density grating of full contrast ($|\rho _{22}-\rho _{11}|=2$) is

33 $$\begin{aligned} |\delta P| = 2T_2d_{12}^2NE/\hbar , \end{aligned}$$

where *N* is the atomic density. Taking into account that $\delta P = \varepsilon _0 \chi E$, we have $|\chi |=2d_{12}^2NT_2/(\hbar \varepsilon _0)$, and therefore the refractive index modification

34 $$\begin{aligned} \delta n \approx \delta \chi /2 = \frac{d_{12}^2T_2N}{\varepsilon _0\hbar }. \end{aligned}$$

**Figure 6:**
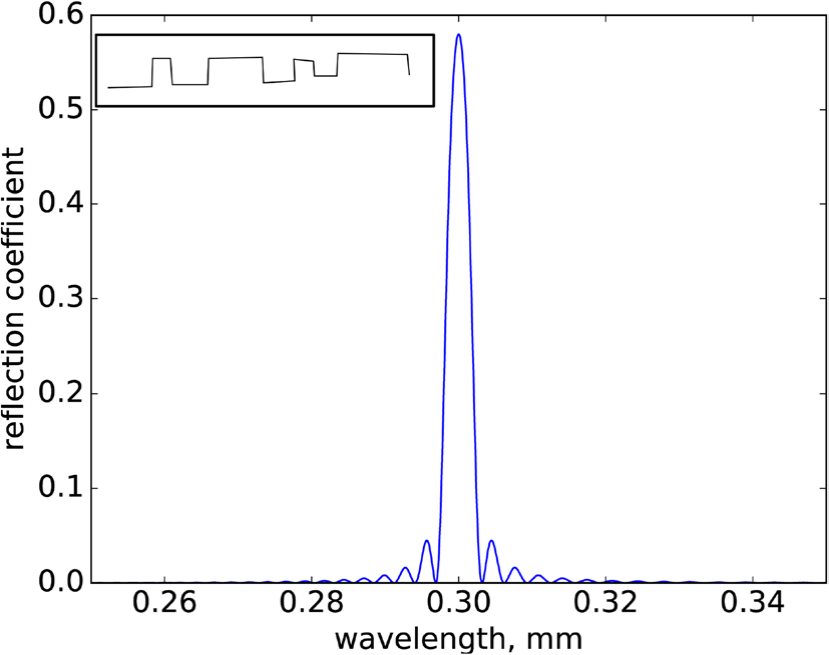
Reflection of a long weak narrow-line probe pulse in dependence on its wavelength from a grating containing 1000 periods ($75\,\mu \hbox {m}$ each) in a medium with 1 bar pressure of the active molecules. The inset shows schematically a CW wave modulated by switching on and off the grating dynamically as a function of time. This figure was created in Phython 2.7, preinstalled with Ubuntu linux 18.04.05 (https://ubuntu.com/).

A refractive index grating is a powerful tool to make highly frequency-selective reflection of THz radiation, fully controllable on the single-cycle (picosecond) time scale. As an example, in Fig. 6 we show the dependence of reflection on a wavelength resulting from a grating containing 1000 oscillation periods, each having the length 75 $\mu$m in a gas with atmospheric pressure consisting of active molecules, which thus creates a density grating with the contrast $\delta n \approx 10^{-3}$. The reflection is calculated using the standard multilevel approach^59^: The ABCD matrix of a single layer of size *h* for the normal incidence is

35 $$\begin{aligned} M(h) = \begin{pmatrix} \cos {(k_0nh)} &{} -\frac{i}{p}\sin {(k_0nh)} \\ -ip\sin {(k_0nh)} &{} \cos {(k_0nh)} \end{pmatrix}, \end{aligned}$$

where $k_0$ is the wavevector in vacuum, *n* is the refractive index, $p=\sqrt{\epsilon }$. For our calculation we have taken $\lambda /4$ layers with interleaving layers of the refractive index $n_1=1-\delta n/2$ and $n_1=1+\delta n/2$. Multiplying the matrices for all layer gives the resulting reflection. Although an analytic expression exists for the whole such product^59^, we find more useful direct numerical multiplication.

The refractive index grating can be used in various ways. The key advantage is a possibility of fast switching on the single-cycle level. An example shown in the inset to Fig. 6 is a fast amplitude modulation of a THz CW wave allowing to encode information into it.

## Conclusions

To summarize, we studied theoretically the possibility of population density grating formation created by subcycle THz pulses in a molecular medium having resonant transitions in THz range. The pulses interact with the molecules coherently and do not overlap in the medium. The amplitude of the grating can be controlled either by tuning the pump amplitude or by an additional pulse. The grating modulation depth is proportional to the square of the THz field amplitude but is also influenced by the coherence times in the medium.

In the case of a multi-level system, using the first order of the perturbation theory in the weak-field approximation, we studied the grating dynamics created by two, three and four subcycle pulses. The possibility of a grating creation and their ultrafast control (erasing and spatial frequency multiplication) in multi-level system excited by subcycle pulses has been shown. A possible scheme of a setup for a grating formation and their control using four counter-propagating pulses on vibrations transitions in a $H_2O$ molecule without overlap in the medium was considered.

Our analysis revealed significant advantages of a grating creation in the THz range with respect to the optical range considered earlier. Because of high dipole moments in the THz range, one needs significantly smaller field amplitudes than in the optical range, of the order of kV/cm. This opens a new opportunity for an ultrafast THz spectroscopy with subcycle THz pulses to determine the resonance frequencies and the corresponding dephasing $T_2$ and relaxation $T_1$ times via diffraction of a weak probe pulse on the induced gratings^12–14^. The investigated phenomenon can be also used for creation of ultrafast beam deflectors and switches on molecular systems in the THz range.

As we have seen, somewhat counter-intuitively, the HO model in the perturbative regime provides lower excitation probabilities of the higher states than, for instance, the atomic potential 1/*r*, and thus the quality of the grating in the molecular case is better than in the case of an atom. This was supported also by non-perturbative simulations with the Bloch equations.

Numerical simulations performed for the parameters of $H_2O$ molecules and analytical results revealed that in the low-field case gratings have a harmonic shape. But in the strong-field case, when the perturbation approach is not valid anymore, the grating shape differs dramatically from the harmonic one. It has complex peak structure, since the level populations in the medium undergo multiple Rabi floppings over the pulse duration.

The obtained results also indicate a  possibility of effective grating creation by quasi-unipolar subcycle THz pulses with respect to bipolar few-cycle ones. This efficient grating creation occurs in spite of the broad spectrum of the subcycle pulses covering many resonances at once, and non-resonant interaction with the molecule transitions. This result opens novel opportunities in ultrafast spectroscopy in the THz range with unipolar half-cycle pulses.
